# Global dataset of soil organic carbon in tidal marshes

**DOI:** 10.1038/s41597-023-02633-x

**Published:** 2023-11-11

**Authors:** Tania L. Maxwell, André S. Rovai, Maria Fernanda Adame, Janine B. Adams, José Álvarez-Rogel, William E. N. Austin, Kim Beasy, Francesco Boscutti, Michael E. Böttcher, Tjeerd J. Bouma, Richard H. Bulmer, Annette Burden, Shannon A. Burke, Saritta Camacho, Doongar R. Chaudhary, Gail L. Chmura, Margareth Copertino, Grace M. Cott, Christopher Craft, John Day, Carmen B. de los Santos, Lionel Denis, Weixin Ding, Joanna C. Ellison, Carolyn J. Ewers Lewis, Luise Giani, Maria Gispert, Swanne Gontharet, José A. González-Pérez, M. Nazaret González-Alcaraz, Connor Gorham, Anna Elizabeth L. Graversen, Anthony Grey, Roberta Guerra, Qiang He, James R. Holmquist, Alice R. Jones, José A. Juanes, Brian P. Kelleher, Karen E. Kohfeld, Dorte Krause-Jensen, Anna Lafratta, Paul S. Lavery, Edward A. Laws, Carmen Leiva-Dueñas, Pei Sun Loh, Catherine E. Lovelock, Carolyn J. Lundquist, Peter I. Macreadie, Inés Mazarrasa, J. Patrick Megonigal, Joao M. Neto, Juliana Nogueira, Michael J. Osland, Jordi F. Pagès, Nipuni Perera, Eva-Maria Pfeiffer, Thomas Pollmann, Jacqueline L. Raw, María Recio, Ana Carolina Ruiz-Fernández, Sophie K. Russell, John M. Rybczyk, Marek Sammul, Christian Sanders, Rui Santos, Oscar Serrano, Matthias Siewert, Craig Smeaton, Zhaoliang Song, Carmen Trasar-Cepeda, Robert R. Twilley, Marijn Van de Broek, Stefano Vitti, Livia Vittori Antisari, Baptiste Voltz, Christy N. Wails, Raymond D. Ward, Melissa Ward, Jaxine Wolfe, Renmin Yang, Sebastian Zubrzycki, Emily Landis, Lindsey Smart, Mark Spalding, Thomas A. Worthington

**Affiliations:** 1 Conservation Science Group, Department of Zoology, University of Cambridge, Cambridge, UK; 2 Biodiversity and Natural Resources Program, International Institute for Applied Systems Analysis (IIASA), Laxenburg, Austria; 3 Department of Oceanography and Coastal Sciences, College of the Coast and Environment, Louisiana State University, Baton Rouge, LA 70803 USA; 4 US Army Engineer Research and Development Center, Vicksburg, MS 39183 USA; 5 Australian Rivers Institute, Centre for Marine and Coastal Research, Griffith University, Nathan, QLD 4117 Australia; 6 DSI-NRF Research Chair in Shallow Water Ecosystems, Institute for Coastal Marine Research, Nelson Mandela University, PO Box 77000, Gqeberha, 6031 South Africa; 7 Department of Agricultural Engineering of the E.T.S.I.A. and Soil Ecology and Biotechnology Unit of the I.B.V., Technical University of Cartagena, 30203 Cartagena, Spain; 8 School of Geography and Sustainable Development, University of St Andrews, KY16 9AL St Andrews, UK; 9 Scottish Association for Marine Science, Oban, Argyll PA37 1QA UK; 10 College of Arts, Law and Education, University of Tasmania, Hobart, Tasmania 7005 Australia; 11 Department of Agricultural, Food, Environmental and Animal Sciences, University of Udine, via delle Scienze 206, Udine, 33100 Italy; 12 Geochemistry and Isotope Biogeochemistry Group, Department of Marine Geology, Leibniz Institute for Baltic Sea Research (IOW), Seestrasse 15, D-18119 Warnemünde, Germany; 13 Marine Geochemistry, University of Greifswald, Friedrich-Ludwig-Jahn Str. 17a, D-17489 Greifswald, Germany; 14 Interdisciplinary Faculty, University of Rostock, Albert-Einstein-Strase 21, D-18059 Rostock, Germany; 15 Department of Estuarine and Delta Systems, Royal Netherlands Institute for Sea Research (NIOZ), 4401 NT Yerseke, The Netherlands; 16 Faculty of Geosciences, Department of Physical Geography, Utrecht University, 3508 TC Utrecht, The Netherlands; 17 Delta Academy Applied Research Centre, HZ University of Applied Sciences, Postbus 364, 4380 AJ Vlissingen, The Netherlands; 18 Tidal Research, Auckland, New Zealand; 19 UKCEH Bangor, Bangor, UK; 20 School of Biology and Environmental Science, University College Dublin, Belfield, Dublin 4, D04 V1W8, Dublin, Ireland; 21 CIMA - Centro de Investigação Marinha e Ambiental, Faro, Portugal; 22 CSIR-CSMCRI, G. B. Marg, Bhavnagar, Gujarat 364002 India; 23 McGill University Department of Geography, Montreal, Canada; 24 Institute of Oceanography - Federal University of Rio Grande, Rio Grande, Brazil; 25 Brazilian Network for Global Change Studies - Rede CLIMA, Rio Grande, Brazil; 26 O’Neill School of Public and Environmental Affairs, Indiana University, Bloomington, USA; 27 University of Georgia Marine Institute, Sapelo Island, Georgia, USA; 28 Centre of Marine Sciences of Algarve, University of Algarve, Faro, Portugal; 29 Univ. Littoral Côte d’Opale, CNRS, Univ. Lille, UMR 8187 - LOG – Laboratoire d’Océanologie et de Géosciences, 32, Avenue Foch, F-62930 Wimereux, France; 30 Institute of Soil Science, Chinese Academy of Sciences, Nanjing, China; 31 School of Geography, Planning Spatial Sciences, University of Tasmania, Launceston, Tasmania 7250 Australia; 32 Department of Environmental Sciences, University of Virginia, 221 McCormick Road, Charlottesville, Virginia 22903 USA; 33 Institute for Biology and Environmental Sciences, Carl von Ossietzky University of Oldenburg, Ammerländer Heerstrase 114-118, D-26129 Oldenburg, Germany; 34 Department of Chemical Engineering, Agriculture and Food Technology, Universitat de Girona, 17003 Girona, Spain; 35 LOCEAN UMR 7159 Sorbonne Université/CNRS/IRD/MNHN, 4 place Jussieu – boite 100, F-75252 Paris, France; 36 IRNAS-CSIC, Avda Reina Mercedes 10, 41012 Seville, Spain; 37 School of Sciences Centre for Marine Ecosystems Research, Edith Cowan University, 270 Joondalup Drive, Joondalup, WA 6027 Australia; 38 Department of Ecoscience, Aarhus University, 8000 Aarhus C, Denmark; 39 School of Chemical Science, Dublin City University, Dublin, Ireland; 40 Department of Physics and Astronomy (DIFA), Alma Mater Studiorum - Università di Bologna, Bologna, Italy; 41 Fudan University, Shanghai, China; 42 Smithsonian Environmental Research Center, Edgewater, USA; 43 School of Biological Sciences, The University of Adelaide, Adelaide, Australia; 44 The Environment Institute, Adelaide, Australia; 45 IHCantabria, Instituto de Hidráulica Ambiental de la Universidad de Cantabria, PCTCAN, 39011 Santander, Spain; 46 School of Resource and Environmental Management, Simon Fraser University, Burnaby, V5A 1S6 Canada; 47 School of Environmental Science, Simon Fraser University, Burnaby, V5A 1S6 Canada; 48 Centro de Estudios Avanzados de Blanes, Consejo Superior de Investigaciones Científicas (CEAB-CSIC), 17300 Blanes, Catalunya Spain; 49 Department of Environmental Sciences, Louisiana State University, Baton Rouge, USA; 50 Zhejiang University, Hangzhou, China; 51 The University of Queensland, St Lucia, Australia; 52 National Institute of Water and Atmospheric Research (NIWA), Hamilton, 3251 New Zealand; 53 School of Environment, University of Auckland, New Zealand, Auckland, 1142 New Zealand; 54 Deakin University, Centre for Marine Science, School of Life and Environmental Sciences, Burwood, Victoria 3125 Australia; 55 MARE - Marine and Environmental Sciences Centre/ARNET - Aquatic Research Network, Department of Life Sciences, University of Coimbra, Coimbra, Portugal; 56 LARAMG – Radioecology and Climate Change Laboratory, Department of Biophysics and Biometry, Rio de Janeiro State University, Rua São Francisco Xavier 524, 20550-013, Rio de Janeiro, RJ Brazil; 57 Faculty of Forestry and Wood Sciences, Czech University of Life Sciences Prague, Kamýcká 129, 165 00, Prague, Czech Republic; 58 U.S. Geological Survey, Wetland and Aquatic Research Center, Lafayette, Louisiana 70506 USA; 59 Department of Zoology and Environment Sciences, University of Colombo, Colombo, 03 Sri Lanka; 60 Soil Science, Hamburg, Germany; 61 Unidad Académica Mazatlán, Instituto de Ciencias del Mar y Limnología, Universidad Nacional Autónoma de México, Mexico City, Mexico; 62 Western Washington University, Bellingham, USA; 63 Elva Gymnasium, Puiestee 2, Elva, 61505 Estonia; 64 National Marine Science Centre, School of Environment, Science and Engineering, Southern Cross University, P.O. Box 157, Coffs Harbour, NSW 2540 Australia; 65 Department of Ecology and Environmental Science, Umeå University, Umeå, Sweden; 66 School of Earth System Science, Institute of Surface-Earth System Science, Tianjin University, Tianjin, China; 67 Departamento de Suelos, Biosistemas y Ecología Agroforestal, MBG sede Santiago (CSIC), Apartado 122, E-15780 Santiago de Compostela, Spain; 68 Department of Environmental Systems Science, ETH Zurich, 8092 Zürich, Switzerland; 69 Department of Life Sciences, University of Trieste, Via L. Giorgieri 10, 34127 Trieste, Italy; 70 Dipartimento di Scienze e Tecnologie Agro-alimentari, Viale G. Fanin, 40 - 40127 Bologna, Italy; 71 Department of Fish and Wildlife Conservation, Virginia Tech, Blacksburg, VA 24060 USA; 72 Centre For Aquatic Environments, University of Brighton, Moulsecoomb, Brighton, BN2 4GJ UK; 73 Institute of Agriculture and Environmental Sciences, Estonia University of Life Sciences, Kreutzwaldi 5, EE-51014 Tartu, Estonia; 74 University of Oxford, Oxford, UK; 75 San Diego State University, San Diego, USA; 76 Center of Earth System Research and Sustainability (CEN), Universität Hamburg, Hamburg, Germany; 77 The Nature Conservancy, Arlington, VA USA; 78 Center for Geospatial Analytics, College of Natural Resources, North Carolina State University, 2800 Faucette Drive, Raleigh, NC 27695 USA; 79 The Nature Conservancy, Strada delle Tolfe, 14, Siena, 53100 Italy

**Keywords:** Carbon cycle, Geochemistry

## Abstract

Tidal marshes store large amounts of organic carbon in their soils. Field data quantifying soil organic carbon (SOC) stocks provide an important resource for researchers, natural resource managers, and policy-makers working towards the protection, restoration, and valuation of these ecosystems. We collated a global dataset of tidal marsh soil organic carbon (MarSOC) from 99 studies that includes location, soil depth, site name, dry bulk density, SOC, and/or soil organic matter (SOM). The MarSOC dataset includes 17,454 data points from 2,329 unique locations, and 29 countries. We generated a general transfer function for the conversion of SOM to SOC. Using this data we estimated a median (± median absolute deviation) value of 79.2 ± 38.1 Mg SOC ha^−1^ in the top 30 cm and 231 ± 134 Mg SOC ha^−1^ in the top 1 m of tidal marsh soils globally. This data can serve as a basis for future work, and may contribute to incorporation of tidal marsh ecosystems into climate change mitigation and adaptation strategies and policies.

## Background & Summary

Tidal marshes are vegetated wetlands formed by herbaceous and woody vascular plants that are present on many of the world’s depositional coastlines and are regularly inundated by tides^1^. While tidal marshes naturally change in extent, anthropogenic pressures (sometimes operating over thousands of years^2^) have greatly accelerated this change in recent decades, degrading their condition globally. Tidal marshes have received considerable attention recently as blue carbon ecosystems, one of a group of ecosystems that have the capacity to capture and store large amounts of soil organic carbon (SOC) over hundreds to thousands of years^3^. Alongside mangroves and seagrasses, they accumulate organic carbon most effectively in their soils where decomposition is slow due to anoxic waterlogged conditions^4,5^. Precise and consistent global-scale information on tidal marsh extent, distribution change, or other ecosystem functions is lacking, highlighting a critical research gap given their potential value for climate change mitigation^6,7^.

Assessments of tidal marsh change have found that previous decades were characterised by extensive losses, with marshes disappearing at a rate of 1–2% per year^8^, leading to a total loss of 67% of tidal marshes over recent centuries^9^. In the period 2000 to 2019, one study estimated a global tidal marsh loss rate of 0.28% per year^10^, while another suggested that marshes have actually marginally increased globally in extent, including vegetation expansion onto existing tidal flats^11^. A new 10 m resolution global map of tidal marsh extent estimates that the ecosystem occupies 52,880 km^2^ (95% confidence intervals: 32,000 to 59,800 km^2^)^12^, similar to previous estimates^13^. These ecosystems continue to be at risk due to direct anthropogenic impacts such as activities that lead to destruction, disturbance, or degradation, sea-level rise, and changes in climate^14^, which negatively impact their ability to retain their stored SOC or accumulate more SOC via carbon sequestration and sediment accretion^15,16^.

The quantification of organic carbon stocks in tidal marsh soils provides critical information to promote the protection, management, and restoration of these natural carbon sinks. Such information, and derived models, may support blue carbon assessments, and enable the incorporation of tidal marsh ecosystems into climate change mitigation and adaptation strategies and policies, including the Nationally Determined Contributions that form a core component of global climate actions. Previous global estimates have averaged values from a few select studies^4,17^, or relied on global datasets that are biassed towards farmland soils^10,18^. There is a clear need for a centralised tidal marsh soil carbon dataset, and to this end the Coastal Carbon Research Coordination Network (CCRCN)^19^ has been collating and publishing core-level datasets. These data are mostly from the United States (U.S.) and have been used to model soil carbon of the Conterminous U.S. tidal marshes^20^. Here, we expand on these efforts by collating site- and core-level tidal marsh SOC data distributed globally.

**Fig. 1:**
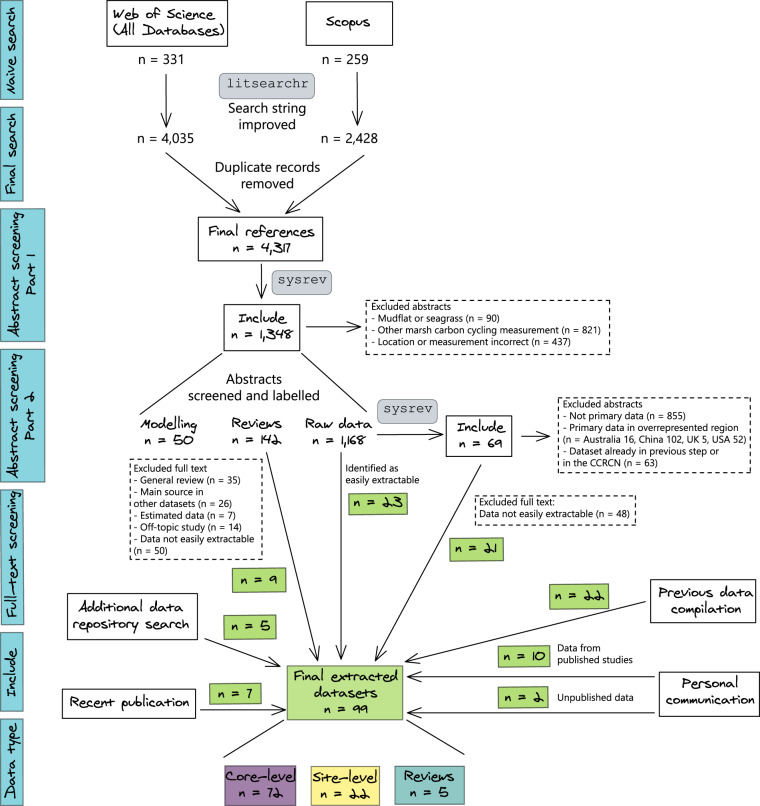
Workflow of the literature search, abstract screening, and dataset generation process for the MarSOC dataset.

We collected data from 99 tidal marsh SOC peer-reviewed and unpublished studies and reformatted the data into a common structure using the R computing environment^21^. Studies were initially identified through a search of the peer-reviewed literature, and data were extracted directly from papers, from data repositories, or through personal communication from authors (Fig. 1). The tidal Marsh Soil Organic Carbon (MarSOC) database^22^ contains 17,454 data points, each with geographic coordinates, collection year, soil depth, and site information (country, site name). The database includes data from 29 countries with an extensive tidal marsh coverage, and over 40% of the data are soil samples deeper than 30 cm. Using these data and the data from the CCRCN^19^, we provide a first order estimate for a globally representative SOC stock value for tidal marshes to 30 cm depth of 79.2 ± 38.1 Mg C ha^−1^ (median ± absolute deviation of the median; n = 26,349), and to 1 m depth of 231 ± 134 Mg C ha^−1^ (n = 39,126). Because marshes can be shallower or deeper than this, region-specific studies should develop their own stock estimates. However, using this value we can estimate an average of 1.22 ± 0.20 Pg C stored in tidal marshes in the upper metre of soil globally.

Generally, carbon content is quantified using an elemental analyzer, but these analyses can incur high costs, particularly in countries where laboratories with this specialised equipment are not easily accessible. Therefore, many studies only record soil organic matter (SOM) content based on Loss On Ignition (LOI). Therefore, to estimate soil organic carbon (SOC) content, a number of equations have been developed to calculate SOC from SOM. For example, Craft and collaborators measured both SOM and SOC from marshes in North Carolina, U.S., and developed an equation for this relationship^23^, which has been used extensively by researchers globally to predict SOC from SOM in wetlands. However, for mangroves, this relationship can change according to the coastal environmental setting^24^, and several studies have generated their own site-specific equations^25–29^. For marshes in the continental U.S., Holmquist and collaborators^20^ developed their own equation using over 1,500 points from 6 studies. Ouyang and Lee^30^ developed a global conversion equation, but they used only a subset of points from each of 11 studies in 4 countries (n = 344). Developing a more globally generalizable equation for tidal marshes is needed for large-scale analyses or as a starting point for new study sites. Within our database there are 17 studies with measurements of both SOM measured via LOI, and SOC measured via elemental analysis, allowing us to present this relationship. We therefore looked to include as many data points distributed globally using data from our database (n = 1,470) and the CCRCN database (n = 3,604), to create a universal conversion equation that spans the diversity of marsh soil types (e.g., minerogenic and organogenic settings) reported in the current literature.

The MarSOC dataset^22^ described here can be used for new global or large-scale estimates of tidal marsh soil organic carbon, and also provide a foundation for additional data collection and collaboration to improve soil organic carbon in tidal marsh estimates, especially from underrepresented areas. The dataset is released for noncommercial use only and is licensed under a Creative Commons Attribution 4.0 International License (CC BY 4.0). All publications that use this database are encouraged to appropriately cite the data and this paper.

## Methods

### Literature search

We compiled the MarSOC dataset from a systematic review of the literature. On 19 January 2022, we searched the title, abstract and keywords in both Scopus and the Web of Science (WoS) All Databases using a naive search string: ((“soil C” OR “soil carbon” OR “soil inorganic carbon” OR “soil organic carbon”) OR (“soil carbon sequestrat*” OR “soil carbon stabiliz*” OR “soil carbon stock*”)) AND (“tidal marsh*” OR “salt marsh*” OR “saltmarsh*”). The search identified 259 studies from Scopus and 331 from the WoS (Fig. 1).

We used the litsearchr R package^31^ to broaden our search terms using keyword co-occurrence networks^31^. All steps can be viewed in the published code with the dataset^22^. This resulted in our final search string: (“blue carbon” OR “carbon accumulation” OR “carbon cycle” OR “carbon dioxide” OR “carbon sequestration” OR “carbon stock*” OR “carbon stor*” OR “organic carbon” OR “organic matter” OR “soil carbon” OR “soil organic carbon” OR “soil organic matter” OR “soil respiration” OR “carbon content” OR “carbon dynamic*” OR “carbon pool*”) AND (“coastal marsh*” OR “coastal salt marsh*” OR “salt marsh*” OR “tidal marsh*” OR “tidal salt marsh*” OR “marsh ecosystem*” OR “marsh soil*” OR “saltmarsh*”).

On 28 January 2022, we searched both Scopus and the WoS All Databases using the final search string mentioned above within the University of Cambridge library account, which includes the following databases: Web of Science Core Collection, BIOSIS Previews, BIOSIS Citation Index, Current Contents Connect, MEDLINE, Zoological Record, Data Citation Index, KCI- Korean Journal Database, SciELO Citation Index, Russian Science Citation Index, and Derwent Innovations Index. This procedure aimed to ensure the inclusion of articles published in languages other than English. We retrieved 4,035 items from WoS and 2,428 from Scopus. We deduplicated the results, giving a total of 4,317 references (Fig. 1), which is tenfold higher than the original naive search.

### Inclusion criteria

The initial and retained articles, with inclusion criteria and additional labels, can be found on our sysrev projects, an open and online tool to screen and label abstracts. In the first sysrev project, we screened the title and abstracts of the 4,317 references to identify those that mentioned soil organic matter or organic carbon in tidal marsh studies. We excluded studies that did not meet these criteria, and separated these into SOC measured in mudflats or seagrasses, other C cycling variables measured in tidal marshes, or studies generally not in tidal marshes or without mention of SOC data. Included studies were labelled as reviews (studies with a general scope, studies with potentially large datasets), modelling (studies with raw data that was used for modelling purposes in that study), and raw data (studies that may contain raw data). Studies could have two tags, such as review studies that included raw data. All studies labelled as “reviews” were retained for full-text assessment, from which we were able to include 9 datasets from tables or the supplementary material. Some of the studies labelled as “raw data” were easily identified as having extractable data (n = 23), such as published datasets (Fig. 1).

To reduce the number of studies requiring full-text screening, from the initial studies tagged “raw data” (n = 1,168), we focused on geographical locations from which we had few datasets (i.e., outside the U.S., U.K., China, and Australia). A second abstract screening with more specific labels was then conducted. We labelled abstracts to identify studies by continent, presence of SOC or SOM data, and inclusion of primary data. A total of 69 studies with primary data in data-poor regions were identified. From these, 21 datasets were extracted or provided by the lead authors on the corresponding papers (Fig. 1).

We searched the SEANOE, PANGAEA, CIFOR, and Marine Scotland Data repositories and found 5 additional studies that fit the inclusion criteria (Fig. 1). We also included data compiled previously for a separate project, which included 12 core-level and 10 site-level published studies. Correspondence with experts in the field led to the inclusion of 10 additional datasets from published studies and 2 from studies that are unpublished or in preparation (see Supplementary Information section I for corresponding sampling methodologies). Finally, data from 7 recent studies published beyond the search date of January 2022 were included. Datasets already held in the Coastal Carbon Research Coordination Network were not included, as our data compilation is intended to be complementary to that research database. The final extracted datasets were from 99 studies (Fig. 1).

### Data acquisition

From the identified studies, when possible, we extracted data (SOM and/or SOC) from the publications’ tables, figures, or supplementary information. When not available, we contacted authors and asked them to contribute their datasets. We downloaded published datasets in repositories from their respective online sources. In total, we extracted data (from tables, supplementary material) from 22 studies, received data via email from 33 studies, and included 22 published datasets from a variety of general (Dataverse, DRYAD, FigShare, Mendeley Data), subject-specific (SEANOE, PANGEA), and country-specific (Environmental Information Data Centre (EIDC), Marine Scotland Data, USGS) repositories. Finally, we appended data from 22 studies from a previous data compilation effort. 

**Fig. 2:**
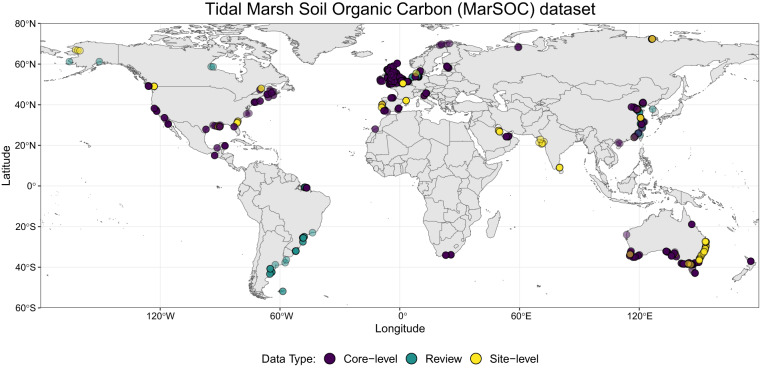
Sample locations coloured by data type (core-level *purple*, review *turquoise*, site-level *yellow*).

In total, we compiled data from 2,329 unique locations (Fig. 2). To be as comprehensive as possible, we included data recorded at the core-level (n = 72 studies^25–28,32–97^), site-level (n = 22^7,98–118^), and from reviews (n = 5^119–123^). This data identification is included in the Data_type column, while the unique ID for each core or plot sampled is reported in the Site_name column (Table 1).

**Table 1:** Description of variables contained in the dataset.

Variable name	Units	Descriptor	Type
Source		Study from which the data was extracted	Character
Original_source		If the source study was a review, the original study of the data	Character
Data_type		Core-level, site-level, or from a review	Character
Site		The name of the site where core(s) were taken	Character
Core		Core ID	Character
Plot		If site-level data, identifier of the site	Character
Site_name		Unique ID per plot or core	Character
Soil_type		Soil type (i.e., peat, sand, silt, mud) when specified	Character
Latitude	Decimal degrees	Geographic coordinate of sample location in WGS84 (N - S)	Numeric
Longitude	Decimal degrees	Geographic coordinate of sample location in WGS84 (E - W)	Numeric
accuracy_flag		Accuracy of geographic coordinate (direct from dataset, averaged, or estimated using Google Earth)	Character
Country		The name of the country where the soil cores were taken	Character
Admin_unit		Administrative unit below country level (Nation, State, Emirate)	Character
Year_collected		The year of the collection. If cores were taken over several years, the year the collection started	Integer
Year_collected_end		If cores were taken over several years, the last year collected	Integer
U_depth_m	Metres	Upper depth of soil core	Numeric
L_depth_m	Metres	Lower depth of soil core	Numeric
Method		Method used to measure organic carbon (%). Elemental analysis (EA), loss-on-ignition (LOI), Walkley Black	Character
Conv_factor		Conversion factor used to convert soil organic matter measured via LOI to organic carbon	Character
OC_perc	%	Soil organic carbon measurement	Numeric
BD_g_cm3	g cm^−3^	Dry bulk density measurement	Numeric
SOM_perc	%	Soil organic matter measurement	Numeric
N_perc	%	Nitrogen (%), if measured alongside C in a CN analyser.	Numeric
Time_replicate		Time replicate for soil sampled more than once a year (summer, winter, month-specific)	Character
Treatment		Site-specific information (control, invaded, univaded, grazed, ungrazed, historic-breach, managed realignment, post-fire)	Character
n_cores		Number of cores in site-level or review measurements, when data available	Integer
SOM_perc_mean	%	Mean of soil organic matter measured (data not at core-level)	Numeric
SOM_perc_sd	%	Standard deviation of the mean of soil organic matter measured	Numeric
OC_perc_mean	%	Mean of soil organic carbon measured (data not at core-level)	Numeric
OC_perc_sd	%	Standard deviation of the mean of soil organic matter measured	Numeric
OC_perc_se	%	Standard error of the mean of soil organic matter measured	Numeric
BD_g_cm3_mean	g cm^−3^	Mean of dry bulk density measured (data not at core-level)	Numeric
BD_g_cm3_sd	g cm^−3^	Standard deviation of the mean of dry bulk density measured	Numeric
BD_g_cm3_se	g cm^−3^	Standard error of the mean of dry bulk density measured	Numeric
OC_from_SOM_our_eq	%	Soil organic carbon estimated from soil organic matter using our equation (Fig. 4)	Numeric
OC_obs_est		Method of OC measurement: “Observed”, “Estimated (study equation)” - OC from LOI with regional eq. (see Conv_factor column), “Estimated (our equation)” - OC from LOI with Eq. 3	Character
OC_perc_final	%	Coalesce of all columns of OC_perc (OC_perc, OC_perc_mean, and OC_from_SOM_our_eq)	Numeric
Notes		Varying sample-specific notes (i.e., flagged outliers)	Character
DOI		Source study DOI URL	Character

**Fig. 3:**
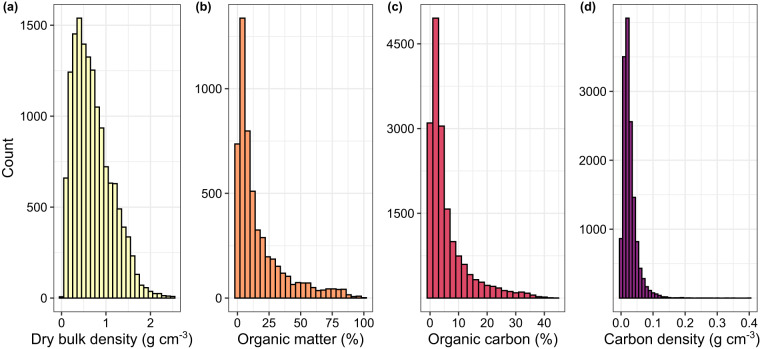
Distribution of data stored in this MarSOC database across all soil depths.

For each data point (i.e., each row), the data include the upper and lower depth of the soil sample, with SOC percent and/or SOM percent (Fig. 3), alongside the method used to determine these values (elemental analyser, Loss-On-Ignition, etc.). Each data point in our dataset also includes geographical coordinates, with a corresponding accuracy flag. If available in the original datasets, dry bulk density (85% of the data) and nitrogen content (15% of the data) were also included. There is also information on the year the sample was collected and the site name and country where the sample was collected, with the name of any finer scale administrative unit if applicable.

Additionally, the data collated here provide the opportunity to calculate an updated and more globally representative average value for the soil organic carbon stock to 1 m depth in tidal marshes. To do so, our database was used with the data from the CCRCN^19^ to maximise the number of points for this calculation (n = 38,945). Using the following equation (Eq. 1), we calculated soil organic carbon density for the subset of soil samples which recorded both a SOC content and measured dry bulk density value (Fig. 3d).

1 $${\rm{SOC}}\;{\rm{density}}\left[{\rm{g}}\;{{\rm{cm}}}^{-{\rm{3}}}\right]={\rm{dry}}\;{\rm{bulk}}\;{\rm{density}}\left[{\rm{g}}\;{{\rm{cm}}}^{-{\rm{3}}}\right]* \left({\rm{SOC}}\left[ \% \right]/100\right)$$

We separated all SOC density samples according to their horizon midpoint into the following soil layer categories: 0–15, 15–30, 30–50, and 50–100 cm (Figure S1). Using all of the measured SOC density values within each of these soil layers (that is, depth interval bins), we calculated the median SOC density value for each layer, along with its absolute deviation. The median was chosen as opposed to the mean due to the skewness of the data (Fig. 3d). We then multiplied this value by the corresponding thickness of each layer, and by 100 to convert grams to megagrams and cubic centimetres to hectares, to get the median SOC stock for each layer (Eq. 2).

2 $${\rm{S}}{\rm{O}}{\rm{C}}\,{\rm{s}}{\rm{t}}{\rm{o}}{\rm{c}}{\rm{k}}[{\rm{M}}{\rm{g}}\,{{\rm{h}}{\rm{a}}}^{-1}]={\rm{S}}{\rm{O}}{\rm{C}}\,{\rm{d}}{\rm{e}}{\rm{n}}{\rm{s}}{\rm{i}}{\rm{t}}{\rm{y}}[{{\rm{g}}{\rm{c}}{\rm{m}}}^{-3}]\ast {\rm{H}}{\rm{o}}{\rm{r}}{\rm{i}}{\rm{z}}{\rm{o}}{\rm{n}}\,{\rm{t}}{\rm{h}}{\rm{i}}{\rm{c}}{\rm{k}}{\rm{n}}{\rm{e}}{\rm{s}}{\rm{s}}[{\rm{c}}{\rm{m}}]\ast 100$$

We then summed these estimated stocks of the four layers to get the estimated total stock to both 30 cm and to 1 m depth. The final estimated value of SOC stock to 30 cm was 79.2 ± 38.1 Mg ha^−1^ (n = 26,239). With an additional 7,204 points located between 30 cm and 50 cm and 5,502 points between 50 and 100 cm, we calculated the stock to 1 m in tidal marsh soils as 231 ± 134 Mg ha^−1^ (median ± median absolute deviation). By using SOC density values from each sample to estimate the density for their respective soil layer (i.e., 0–15, 15–30, 30–50, and 50–100 cm), all data points were used in the stock calculation without needing to extrapolate. To get a more refined estimate of global tidal marsh soil carbon storage, it is possible to multiply this stock value by the tidal marsh area estimate of 52,880 km^2^ (95% CI: 32,000 to 59,800 km^2^) from the recent globally consistent extent map^12^. This gives us a global estimate of tidal marsh soil carbon of around 1.22 ± 0.20 Pg C in the top metre of soil, which is lower than previous estimates^17^. However, we acknowledge that this is a general estimate, and that a study using machine learning and environmental predictors to estimate SOC at a finer scale would give a more appropriate and accurate spatial representation of SOC stocks across the world’s coastal marshes. We also acknowledge that tidal marsh soils in different regions may be more shallow, or deeper than 1 m, so we recommend that regional studies develop their own carbon stock estimates.

### Global conversion factor

**Fig. 4:**
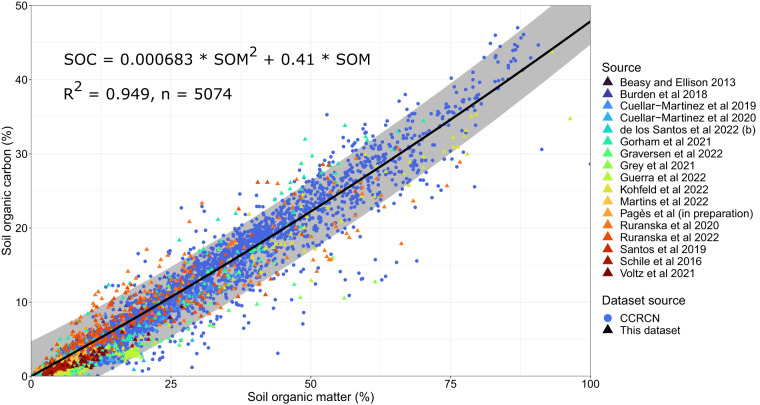
Data points with both soil organic matter and soil organic carbon values, used to calculate the conversion equation for SOM to SOC (solid *black* line, with prediction intervals in *grey*). Data extracted from the Coastal Carbon Research Coordination Network (CCRCN)^19^ are shown in *circles*, and values from this dataset are shown in *triangles*.

**Fig. 5:**
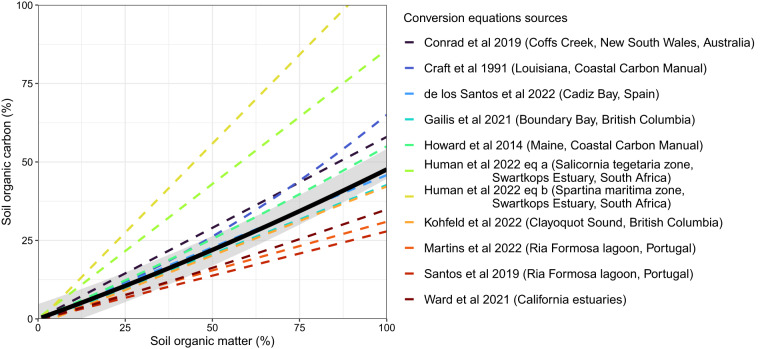
Soil organic matter to soil organic carbon conversion relationships developed by different sources, along with the region, site, or species zone from which these were developed (equations detailed in Table S1). Our conversion equation is a solid *black* line, with prediction intervals in *grey*.

To create our conversion factor between SOC and SOM, we identified 17 studies in which, both SOM and SOC were measured. While data from the CCRCN is not included in our final dataset, we did include all data with both SOM and SOC measurements from the CCRCN^19^ to create the conversion factor equation. Thus, we included 18 studies^124–128^ from the CCRCN^129–141^ and 17 studies from our dataset to investigate the SOM to SOC relationship (Fig. 4). A further 10 studies, in which the authors developed their own conversion factor to convert SOM to SOC (Fig. 5), were selected for comparison.

To model SOC from SOM, we used the nls() and the lmer() functions in R to fit linear and quadratic models with an intercept fixed to 0, and included the study ID as a random effect. Based on Akaike’s Information Criteria, testing model parsimony relative to explanatory power, the best fitting model was a quadratic function with study ID as a random effect (Eq. 3; Fig. 4, R^2^ = 0.949, n = 5074).

3 $${\rm{SOC}}\left[ \% \right]=\left(0.000683\pm 0.00563\right)\ast {\rm{SOM}}{\left[ \% \right]}^{2}+\left(0.410\pm 0\right)\ast {\rm{SOM}}\left[ \% \right]$$

This can be compared to 16 studies from our literature search that used a variety of conversion factors (Table S1). We also fitted a quadratic model to each of the individual studies presented in Fig. 4, used to generate the general equation (Figure S2). We found that many of the study-specific quadratic equations were significantly different to the overall equation (Table S2), showing that there is high variability in the relationship between SOM and SOC between each study. While site-specific conversion equations will always be desirable, our general model captures a range of coastal tidal marsh types distributed across the climatic, oceanographic, and geomorphic gradients with applications to regional or larger-scale studies. Our equation lies amongst the other conversion equations (Fig. 5), and estimates less organic carbon from organic matter than the commonly used Craft^23^ equation or the second equation presented in Blue Carbon Initiative handbook^142^, which used data from Maine. Our dataset can be used to analyse the uncertainty in how these different equations affect the calculation of a C stock for soils. For example, the uncertainty may be different for varying levels of soil organic matter, or for marshes with different coastal geomorphologies or soil type, which may influence the relationship between SOM and SOC^24,143^. It can also be used to estimate soil carbon stocks in tidal marshes for varying soil depths and using different methods, such as extrapolating cores to 1 m or confining the analysis to the topsoil. Finally, the data can serve as a basis for future work integrating other soil variables, such as soil total inorganic carbon, particulate organic and inorganic carbon, as well as isotope measurements.

## Data Records

The data and code used in the methods described above are archived in a Zenodo repository^22^. This is a static copy of the data peer-reviewed in 2023, which is a release from the dynamic Github repository https://github.com/Tania-Maxwell/MarSOC-Dataset. The data is currently being incorporated into the CCRCN Atlas.

The repository is formatted in the following structure:

- Maxwell_MarSOC_dataset.csv: .csv file containing the final dataset. The data structure is described in the metadata file. It contains 17,454 records distributed amongst 29 countries.
- Maxwell_MarSOC_dataset_metadata.csv: .csv file containing the main data file metadata (equivalent to Table 1).
- data_paper/: folder containing the list of studies included in the dataset, as well as figures for this data paper (generated from the following R script: ‘reports/04_data_process/scripts/04_data-paper_data_clean.R’).
- reports/01_litsearchr/: folder containing.bib files with references from the original naive search, a .Rmd document describing the litsearchr analysis using nodes to go from the naive search to the final search string, and the.bib files from this final search, which were then imported into sysrev for abstract screening.
- reports/02_sysrev/: folder with.csv files exported from sysrev after abstract screening. These files contain the included studies with their various labels.
- reports/03_data_format/: folder containing all original data, associated scripts, and exported data.
- reports/04_data_process/: folder containing data processing scripts to bind and clean the exported data, as well as a script testing the different models for predicting soil organic carbon from organic matter and finalising the equation using all available data. A script testing and removing outliers is also included.

## Technical Validation

For consistency and to validate the inclusion criteria, the literature search and screening was conducted in a two-part process that included a repeated evaluation by different co-authors. All SOC and SOM values were extracted from numerical sources (tables, supplementary tables, or published datasets). The distribution of all quantitative variables was verified visually by two authors, and the following outliers were flagged: 1) SOC, SOM, and dry bulk density values greater than the sum of 2.2x the interquartile range plus the 95% percent quantile^144^ of this dataset combined with the CCRCN dataset, 2) SOM values greater than 100, and 3) SOC values greater than SOM values, which may have been due to incomplete removal of water prior to LOI or due to incomplete removal of carbonates prior to SOC measurements. These values were removed from all calculations but remain in the dataset with an outlier flag in the “Notes” column of the dataset. In total, this represented less than 1% of data removal. These operations and the distribution of all variables (Fig. 3) can be found in the script 02_outliers.R.

## Usage Notes

This data descriptor manuscript and dataset was peer reviewed in 2023 based on a targeted search of the data available at the time. This compilation of 99 published and unpublished tidal marsh soil carbon datasets can be used to answer multiple research questions. First, the MarSOC dataset can be used to support large-scale models of soil carbon in tidal marshes and improve global estimates of carbon stored in these coastal ecosystems. Different drivers of soil carbon at the landscape-scale can be investigated, such as the influence of coastal geomorphology. In addition, our database serves as a baseline for targeted ecosystem design outcomes and restoration of degraded tidal marshes.

### Supplementary information

Supplementary Information

## Data Availability

The code to format and process data was developed in R computing environment and is freely available in the Zenodo repository^22^. This is a static copy of the data peer-reviewed in 2023, which is a release from the dynamic Github repository https://github.com/Tania-Maxwell/MarSOC-Dataset. When using data from this dataset please cite this publication, along with the original sources. Both dataset and code are available under a Creative Commons License (CC-BY).
