# Supplementary material for: Global dataset of soil organic carbon in tidal marshes
Source: Sci Data. 2023 Nov 11;10:797. doi: 10.1038/s41597-023-02633-x (PMC10640612; doi:10.1038/s41597-023-02633-x)
Supplement: Supplementary file 1 — Supplementary Information [file 41597_2023_2633_MOESM1_ESM.pdf]

Supplementary Information

Supplement to Maxwell, T. *et al.* Global dataset of soil organic carbon in tidal marshes. *Scientific Data* (2023).

Table of Contents

I. Methods for unpublished data ... 1

Pagès et al in prep ... 1

Serrano et al unpublished ... 2

II. Tables and Figures ... 3

III. Supplementary References ... 7

I. Methods for unpublished data

Pagès et al in prep

Site description and sampling

In May-July 2017, three replicate soil cores were collected from three salt marshes in three estuaries in West Wales, United Kingdom. All marshes were similar in terms of elevation, extent, geomorphology (i.e., estuarine marshes), position within the estuary (i.e., situated in the mid part of the estuary, sheltered from the ocean) and land use (i.e., light to moderately grazed). All cores were extracted at similar elevations relative to mean sea level (i.e., 1.4-2.0 m above mean sea level, obtained using a Leica differential GPS). Replicate cores were collected within tens of metres of each other. The cores were sampled by manual percussion and rotation using PVC pipes and following the guidelines in the Blue Carbon Manual[1]. Compression during coring was assessed by measuring the length of the core protruding from the soil surface inside and outside the core[1]. All results reported in this study refer to the decompressed depths (cm). Upon extraction, cores were sealed at both ends, transported to the laboratory and stored at 4°C until processing.

Laboratory analysis

Cores were sliced at 2 cm-thick resolution for their entire length. Each slice was dried at 60°C until constant dry weight (DW) to estimate dry bulk density, homogenized and divided into two sub-samples. One subsample was analysed for soil grain-size, using a Malvern Particle Sizer 2000, after organic matter digestion with hydrogen peroxide (conducted at the Geography Science Laboratories of the Department of Geography, University of Cambridge). Another sub-sample was ground in an agate mill and analysed for: organic carbon, loss on ignition, ^{13}C, ^{15}N and nitrogen content. For organic carbon (Corg) analysis, about 5 g of ground sample

was acidified with 4% HCl to remove inorganic carbon, centrifuged (3400 rpm during 5 min), and the supernatant with acid residues carefully removed by pipette, avoiding resuspension. The sample was then washed with Milli-Q water, centrifuged and the supernatant again removed. The residual samples were re-dried (60°C until constant weight) and encapsulated in tin capsules. Corg was analysed using a Costech Elemental Analyzer interfaced to a Thermo-Finnegan Delta V Isotope RatioMass Spectrometer at UH-Hilo Analytical Laboratory. The accuracy of the analysis of the Standard reference material NIST 8704 (Buffalo River Sediment) was ≤1%. The Corg content (%) is reported for the bulk (pre-acidified) samples.

Reference

Pagès, J. F., McKinley, E., Ladd, C. J. T., Leiva-Dueñas, C., Piñeiro-Juncal, N., Mateo, M. A. & Skov, M. W. (in prep). *Resilience of saltmarsh carbon sequestration to ecosystem transitions*.

Serrano et al unpublished

Site description and sampling

The three unpublished cores “OS MCD1”, “OS MCD2”, and “OS MCD3” were sampled on Torrens Island in an estuary near Adelaide, Australia. Core locations were recorded with a GPS. The soil sampling procedure follows that presented by the study Gorham et al., 2021[2]. Briefly, the soil cores were sampled by manually hammering PVC pipes with a 7.5 cm inner diameter and a length of 1.5 m into tidal marsh soil. Compaction was minimised by hammering softly and rotating the corer often. The difference in surface soil elevation on the inside and outside of the core was measured and recorded to assume linear downcore soil compaction[3]. The samples were kept at 4°C until further analysis.

Laboratory analysis

Back in the laboratory, the cores were opened lengthwise and sliced at 1 cm intervals. Bulk density was calculated after drying the soil samples at 70°C. Soil samples were then milled to a fine powder. The subsequent soil sample analysis procedure was the following: digestion of carbonates with 4% HCl, centrifugation, decanting the supernatant, rinsing with milliQ water, centrifugation, decanting, and drying at 70°C prior to encapsulation. The organic carbon was then measured using an elemental analyzer coupled with a mass spectrometer.

Reference

Serrano, O. *et al.* (unpublished). *Soil carbon cores from Australian saltmarshes*.

II. Tables and Figures

Source	Conversion factor	Equation source	Final equation used	n
Burke et al 2022	SOC = 0.4*SOM + 0.0025*(SOM^2)	Craft et al 1991 (general)	Our equation	157
Conrad et al 2019	SOC = 0.58*SOM	Brown et al. 2016 (region-specific)	Study equation	33
Hatje et al 2023	SOC = 0.47*SOM + 0.0008	Derivative of Howard et al 2014 (general)	Our equation	251
de los Santos et al 2022 (a)	SOC = 0.2822*SOM -0.3401	Fitted to data from Santos et al 2019 (site-specific)	Study equation	39
de los Santos et al 2022 (b)	SOC = 0.461*SOM - 0.266	Fitted to data (site-specific)	Study equation	9
Gailis et al 2021	SOC = 0.44*SOM - 1.33	Fitted to data and Chastain 2017 (region-specific)	Study equation	22
Gispert et al 2020	SOC = SOM/1.724	Walkley-Black method (specific to method)	Study equation	6
Gispert et al 2021	SOC = SOM/1.724	Walkley-Black method (specific to method)	Study equation	4
Gu et al 2020	SOC = 0.4*SOM + 0.0025*(SOM^2)	Craft et al 1991 (general)	Our equation	147
Human et al 2022	SOC = 0.8559*SOM + 0.1953	Fitted to data (species-specific)	Study equation	412
Human et al 2022	SOC = 1.1345*SOM - 0.8806	Fitted to data (species-specific)	Study equation	412
Kohfeld et al 2022	SOC = 0.44*SOM - 1.80	Fitted to data (site-specific)	Study equation	704
Martins et al 2022	SOC = 0.3102*SOM - 0.066	Fitted to data (site-specific)	Study equation	127
Perera et al 2022	SOC = 0.47*SOM	From different region (Baustian et al 2017)	Our equation	16
Ward 2020	SOC = 0.47*SOM + 0.0008*(SOM^2)	Howard et al 2014 (general)	Our equation	10
Ward et al 2021	SOC = 0.22*(SOM^1.1)	Fitted to data (site-specific)	Study equation	192
Wollenberg et al 2018	SOC = 0.40*SOM + 0.0025*(SOM^2)	Craft et al 1991	Our equation	28

Table S1 Transfer equations used in different studies to calculate soil organic carbon (SOC) from soil organic matter (SOM), along with number samples (n) for which the transfer equation was used to estimate SOC from measured SOM (i.e., not the number of samples with both SOM and SOC used to develop the equations).

Study	Res.Df	Res.Sum Sq	Df	Sum Sq	F value	p-value
All studies	5072	29231.576	NA	NA	NA	NA
Beasy and Ellison 2013	11	0.425	5061	29231.151	149.616	<0.0001
Burden et al 2018	29	3.935	-18	-3.51	5.052	0.004
CCRCN	3602	20209.813	-3573	-20205.878	41.678	<0.0001
Cuellar-Martinez et al 2019	62	30.995	3540	20178.818	11.402	<0.0001
Cuellar-Martinez et al 2020	52	53.675	10	-22.68	-2.197	1
Gorham et al 2021	223	796.103	-171	-742.429	4.206	<0.0001
Graversen et al 2022	168	297.533	55	498.57	5.118	<0.0001
Grey et al 2021	13	49.28	155	248.253	0.423	0.994
Guerra et al 2022	75	8.259	-62	41.021	-0.175	1
Kohfeld et al 2022	85	520.708	-10	-512.449	465.345	<0.0001
Pagès et al (in preparation)	149	26.096	-64	494.612	-1.262	1
Ruranska et al 2020	153	1687.387	-4	-1661.291	2371.348	<0.0001
Ruranska et al 2022	196	913.266	-43	774.121	-1.632	1
Santos et al 2019	14	1.554	182	911.712	45.127	<0.0001
Martins et al 2022	67	6.151	-53	-4.597	0.781	0.749
de los Santos et al 2022 (b)	16	1.278	51	4.873	1.196	0.359
Schile et al 2016	93	24.575	-77	-23.297	3.788	0.002
Voltz et al 2021	30	11.13	63	13.446	0.575	0.967

Table S2 Analysis of variance table comparing the study-specific models to the general model, which corresponds to the quadratic equation relationship between soil organic matter and soil organic carbon, presented in Figure S2.

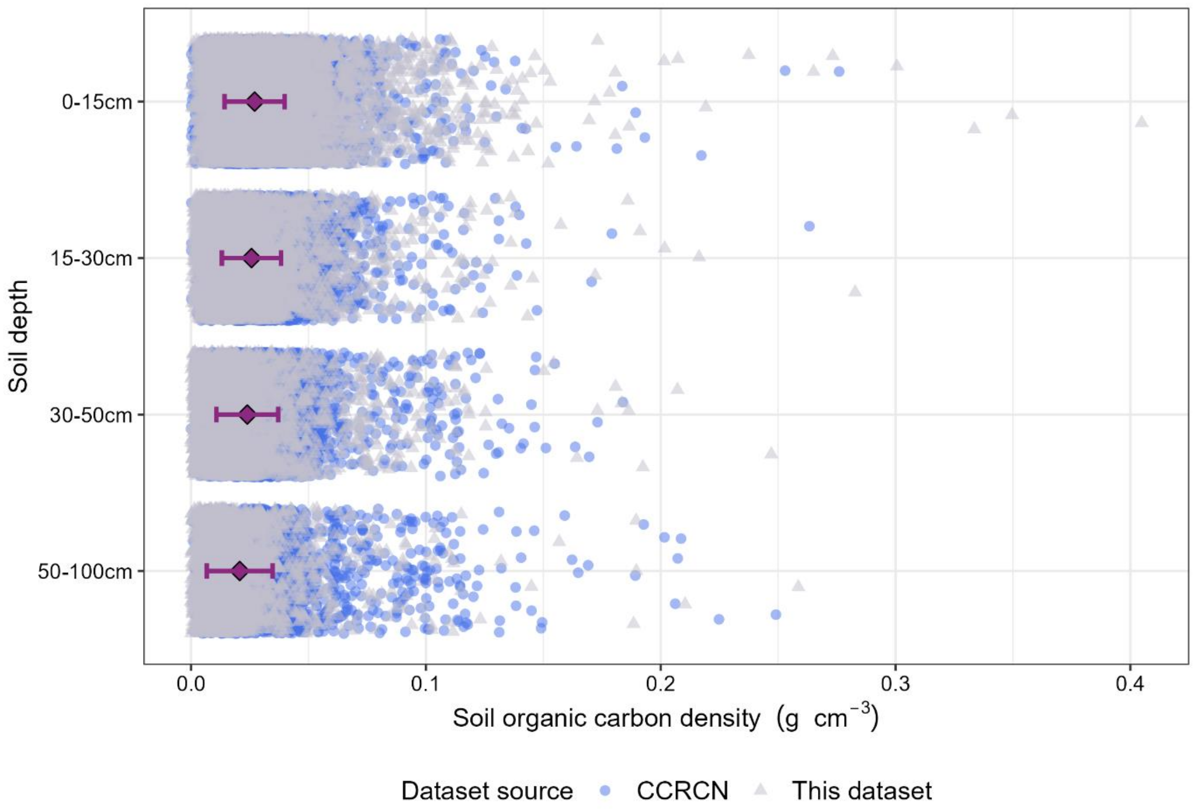

Fig. S1 Soil organic carbon density (kg m^{-3}) from this dataset (grey triangles) and from the Coastal Carbon Research Coordination Network (CCRCN) in blue circles, binned into four horizon depths, according to the sample layer mid-point: 0-15, 15-30, 30-50, 50-100 cm. Values in the purple *diamonds* correspond to the median for each horizon bin, and error bars are the absolute deviation of the median.

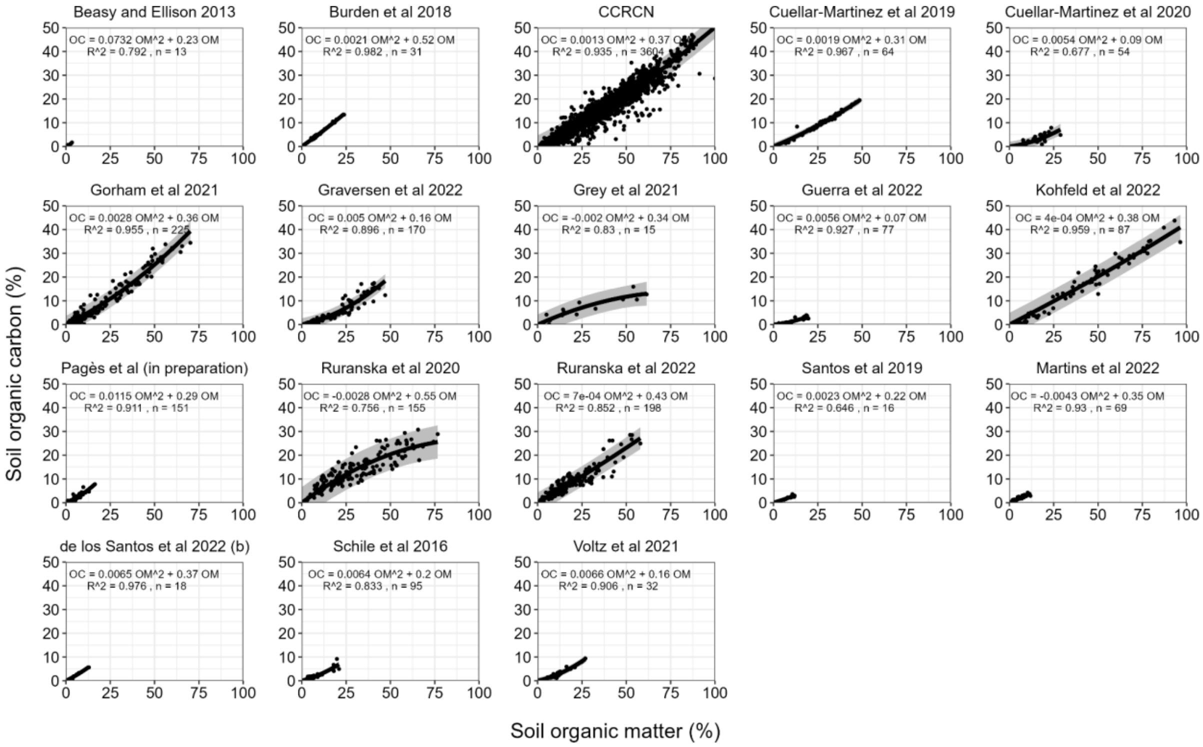

Fig. S2 One plot for each study located in Figure 4 of the manuscript, with data points with both soil organic matter and soil organic carbon values, used to calculate a study-specific conversion equation for SOM to SOC (solid *black* line, with prediction intervals in *grey*).

III. Supplementary References

1. Howard, J., Hoyt, S., Isensee, K., Telszewski, M. & Pidgeon, E. *Coastal blue carbon: methods for assessing carbon stocks and emissions factors in mangroves, tidal salt marshes, and seagrasses*. https://cgspace.cgiar.org/handle/10568/95127 (2014).
2. Gorham, C., Lavery, P., Kelleway, J. J., Salinas, C. & Serrano, O. Soil Carbon Stocks Vary Across Geomorphic Settings in Australian Temperate Tidal Marsh Ecosystems. *Ecosystems* **24**, 319–334 (2021).
3. Glew, J. R., Smol, J. P. & Last, W. M. Sediment core collection and extrusion. *Track. Environ. Change Using Lake Sediments Basin Anal. Coring Chronol. Tech.* 73–105 (2001).
4. Troels-Smith, J. *Characterization of unconsolidated sediments*. (Reitzels Forlag, 1955).
5. Dadey, K. A., Janecek, T. & Klaus, A. Dry-bulk density: its use and determination. in *Proceedings of the Ocean Drilling Program, Scientific Results* vol. 126 551–554 (National Science Foundation, & Joint Oceanographic Institutions Incorporated …, 1992).
6. Natali, C., Bianchini, G. & Carlino, P. Thermal stability of soil carbon pools: Inferences on soil nature and evolution. *Thermochim. Acta* **683**, 178478 (2020).
7. Smeaton, C., Hunt, C. A., Turrell, W. R. & Austin, W. E. Marine sedimentary carbon stocks of the United Kingdom's exclusive economic zone. *Front. Earth Sci.* 50 (2021).
8. DIN 19539. 2015-08, Investigation of solids Temperature dependent differentiation of Total Carbon (TOC400, ROC, TIC900).
